# Use of male-to-female sex reversal as a welfare scoring system in the protandrous farmed gilthead sea bream (*Sparus aurata*)

**DOI:** 10.3389/fvets.2022.1083255

**Published:** 2023-01-09

**Authors:** Paul G. Holhorea, Alicia Felip, Josep À. Calduch-Giner, Juan Manuel Afonso, Jaume Pérez-Sánchez

**Affiliations:** ^1^Nutrigenomics and Fish Growth Endocrinology Group, Institute of Aquaculture Torre de la Sal, CSIC, Castellón, Spain; ^2^Group of Fish Reproductive Physiology, Institute of Aquaculture Torre de la Sal, CSIC, Castellón, Spain; ^3^Aquaculture Research Group, Institute of Sustainable Aquaculture and Marine Ecosystems (IU-ECOAQUA), University of Las Palmas de Gran Canaria, Las Palmas, Spain

**Keywords:** protandrous fish, sex reversal, sex steroids, welfare scoring, plant-based diets, nutrition and genetics interactions

## Abstract

Gilthead sea bream is a highly cultured marine fish throughout the Mediterranean area, but new and strict criteria of welfare are needed to assure that the intensification of production has no negative effects on animal farming. Most welfare indicators are specific to a given phase of the production cycle, but others such as the timing of puberty and/or sex reversal are of retrospective value. This is of particular relevance in the protandrous gilthead sea bream, in which the sex ratio is highly regulated at the nutritional level. Social and environmental factors (e.g., contaminant loads) also alter the sex ratio, but the contribution of the genetic component remains unclear. To assess this complex issue, five gilthead sea bream families representative of slow/intermediate/fast growth were grown out with control or a plant-based diet in a common garden system from early life to the completion of their sexual maturity in 3-year-old fish. The plant-based diet highly enhanced the male-to-female sex reversal. This occurred in parallel with the progressive impairment of growth performance, which was indicative of changes in nutrient requirements as the result of the different energy demands for growth and reproduction through development. The effect of a different nutritional and genetic background on the reproductive performance was also assessed by measurements of circulating levels of sex steroids during the two consecutive spawning seasons, varying plasma levels of 17β-estradiol (E_2_) and 11-ketotestosterone (11-KT) with age, gender, diet, and genetic background. Principal component analysis (PCA) of 3-year-old fish displayed a gradual increase of the E_2_/11-KT ratio from males to females with the improvement of nutritional/genetic background. Altogether, these results support the use of a reproductive tract scoring system for leading farmed fish toward their optimum welfare condition, contributing to improving the productivity of the current gilthead sea bream livestock.

## Introduction

Fish farming has evolved as one of the most sustainable production sectors because of its high feed conversion efficiency and its lower carbon footprint when compared with other animal production systems (1). Nonetheless, aquaculture production is becoming more intensified to meet the increased global demand for fish protein aquaculture (2). It is, thereby, important to encompass the development of aquaculture with novel and stricter criteria of welfare for the simultaneous improvement of aquaculture productivity and welfare of farmed fish (3, 4). Certainly, important research efforts are now conducted within the AquaIMPACT H2020 project for integrating information from fish breeding and nutrition to promote the production of healthier and more robust fish with higher phenotypic plasticity to cope with a challenging environment. This includes the use of gut microbiota as a reliable criterion to evaluate the success of selective breeding for improving the performance and competitiveness of European aquaculture (5, 6). At the same time, novel fish feed formulations, and epigenetic and behavioral approaches are widely applied to assure a more ethical and sustainable aquaculture production with the increase of water temperature and hypoxia as major environmental problems in coastal marine ecosystems (7–9). Thus far, fish welfare assessment is still in the infancy state due to the limited understanding of the diverse fish species' welfare-relevant biology (10). However, several benchmarking systems on key performance indicators (KPIs) based on growth performance, survival rates, and external tissue damage (skin/fin erosion) have been currently validated in salmon, but also in Mediterranean fish species, to ensure that farmed fish are not far from their optimum welfare (11–14). Otherwise, behavioral indicators are becoming especially useful for alerting farmers that something is potentially wrong and warrants investigation before significant welfare issues can occur (15, 16). In any case, the best monitoring solution, especially those based on telemetry techniques and bio-loggers for tracking swimming activity and/or heart or breathing rates, highly depends on the asked question, species biology, and culture system (17).

A common feature of welfare indicators is their accuracy for a given time and culture condition, reflecting immediacy rather than a historical background. However, the success of reproductive performance, measured by means of fecundity, puberty onset, and sex reversal, can also have a high value from a retrospective point of view, as it is the end point of a complex cascade of developmental events that encompass a wide range of biotic and abiotic factors (18–22). In particular, the sex ratio in gonochoristic fish tends to be balanced in optimal culture conditions (23), although it can be affected by chemicals (24) and other environmental factors such as rearing density, temperature, pH, oxygen, and diet composition (25–29). Similarly, sex reversal in hermaphrodite species, such as in the protandrous gilthead sea bream, is socially controlled and endocrine-regulated by the circulating levels of estradiol (E_2_) and 11-ketotestosterone (11-KT) (30), and intriguingly the exposure to synthetic estrogens (e.g., 17α-ethynylestradiol) prevents the male-to-female sex reversal (31). Stress may also influence the onset of sex change through the mediation of cortisol, although the exact mechanisms in which it may act as a mediator in sex change remain to be fully established (32). Otherwise, puberty onset is determined by genetic factors and controlled by the nutritional status and/or the body's growth (33). Thus, similar to what occurs in humans, better welfare conditions for fish entail an increase in their growth before reaching their first sexual maturation (34, 35). However, early puberty, in particular in males, occurs in several species kept under aquaculture conditions and is often associated with a final growth retardation or health risks (36, 37). Moreover, the age of puberty can be controlled in farmed fish by selective breeding and feeding level (38–40), and a recent gilthead sea bream study stated that plant-based diets have the potential to alter the sex steroid profile during the pre-spawning and spawning period, promoting the enhanced male-to-female sex reversal when the presence of powerful functional females is compromised by the diet (41).

Taking into account all the above findings, we had herein a double objective: (i) to assess how the male-to-female sex reversal is affected by nutrition and genetics in the protandrous gilthead sea bream and (ii) to provide new insights into the use of male-to-female sex reversal and population sex ratio as a reliable best practice framework for animal welfare certification of a highly cultured farmed fish in all the Mediterranean basin. The rationale for this procedure is that the complex balance of environmental variables that regulate animal welfare conditions can also affect sex change in sequential hermaphrodites. To pursue this issue, sex reversal was monitored in fish families with different nutritional backgrounds and different heritable growth within the PROGENSA^®^ selection program (42), which co-selected among other traits with changes in gut microbiota composition and metabolic plasticity (5, 43), as well as swimming performance and aerobic scope (44, 45).

## Materials and methods

### Diets

Two extruded diets were formulated and produced by BioMar (BioMar Process Innovation Technical Center, Brande, Denmark), at a range of pellet sizes corresponding to the respective fish size as fish grew (i.e., 1.9, 3, 4.5, and 6.5 mm). Both diets were isonitrogenous, isolipidic, and isoenergetic and met all known nutritional requirements of gilthead sea bream. Fish meal (FM) was included at 23% in the control diet (D1) and at 3% in the experimental diet (D2). The addition of fish oil (FO) was 14.1% for D1, and 3.9% for D2 with the replacement of rapeseed oil, decreasing EPA+DHA content from 3.8 to 1.02%. Lysine, methionine, choline, lecithin, and monocalcium phosphate were added to D2 to reach D1 levels (Supplementary Table S1).

### Experimental setup and sample collection

Broodstock crossings of eight (two females and six males) and five (three females and two males) fish from the gilthead sea bream PROGENSA^®^ selection program rendered sixteen families with differences in heritable growth, as described elsewhere (42). Briefly, juvenile fish of these families, previously genotyped by DNA fin analysis, were individually tagged (dorsal muscle) with passive integrated transponders (PITs) (ID-100A 1.25 Nano Transponder, Trovan, Madrid, Spain) and maintained in a common garden system fed D1 or D2 diets in replicate 3,000-L tanks under the natural photoperiod and temperature conditions (latitude 40° 5′ N; 0° 10′ E) at the Institute of Aquaculture Torre de la Sal (IATS), over the course of a 12-month feeding trial (September 2017 to September 2018). At this end, five families were selected by their growth trajectories during this period as a representative of fast growth (e5e2, e6e2; 158 and 49 individuals, respectively), intermediate growth (c2c7, e4e1; 91 and 174 individuals, respectively), and slow growth (c4c3; 60 individuals), and were distributed at similar family in the common garden system. Growth performance and reproductive status were assessed in these families until the completion of sexual maturation in 3-year-old fish (December 2020).

Over the course of the entire trial, the concentration of water oxygen was always higher than 80% saturation. Fish were fed by automatic feeders 1–2 times per day and 3–7 days per week according to fish size and season, with the ratio adjusted weekly to a level close to satiation. The final rearing density was 19–20 kg/m^3^. Fish body weight and body length were measured individually using an FR-200 FishReader W (Trovan, Madrid, Spain) at different monthly intervals during the first (Age +1), second (Age +2), and third year (Age +3) of the production cycle. At the time of a maximum number of spermiating fish (December), overnight-fasted fish were anesthetized with 100 mg/L MS-222 (Sigma, Saint Louis, MO, USA) for blood extraction and sexing by stripping. It is a non-lethal and accurate sexing method at this time and developmental stage as almost all males are fluent by stripping, in coincidence with the annual peak of E_2_ in females and 11-KT in males that resulted in a minimum presence (<5%) of intersex fish (41). Blood was collected (100 fish/diet for Age +2 fish, 150 fish/diet for Age +3 fish) from caudal vessels using heparinized syringes and centrifuged at 3,000 × g for 20 min at 4°C, and plasma aliquots were stored at −20°C until sex steroid analyses.

All procedures were approved by the Ethics and Animal Welfare Committee of IATS and CSIC. They were carried out in the IATS's registered aquaculture infrastructure facility (code ES120330001055) in accordance with the principles published in the European Animal Directive (2010/63/EU) and Spanish Laws (Royal Decree RD53/2013) for the protection of animals used in scientific experiments.

### Sex steroids

Quantification of plasma sex steroids was performed by enzyme immunoassays (EIAs) as described by Rodríguez et al. (46) for 11-KT and by Molés et al. (47) for E_2_. Briefly, steroids were extracted from 100 μl plasma in 1 ml methanol and supernatants were dried and reconstituted in EIA buffer (0.1 M potassium phosphate, pH 7.4 containing 0.01% sodium azide, 0.4 M NaCl, 0.001 M EDTA, and 0.1% BSA). Steroid standards were purchased from Sigma-Aldrich. Mouse anti-rabbit immunoglobulin monoclonal antibody (Ab), rabbit steroid Abs (T-Ab, 11-KT-Ab, and E_2_-Ab), and enzymatic tracers [steroid acetylcholinesterase (AChE) conjugates: T-AchE, 11-KT-AChE, and E_2_-AChE] were obtained from Vitro S.A. (Sevilla, Spain). Samples and standard curves of 11-KT (0.0001–1.0 ng/ml) and E_2_ (0.005–9.0 ng/ml) were run in duplicate. Optical density was read at 405 nm using a microplate reader (Bio-Rad 3550). The inter-assay coefficients of variation at 50% of binding were 5.02% (*n* = 10) with a 0.88 slope for 11-KT and 5.97% (*n* = 10) with a 0.68 slope for E_2_.

### Statistical analysis

Statistical analysis was performed using SigmaPlot version 14.0 (Systat Software, San Jose, CA, USA) with all *P*-values set to 0.05 for significance determination. Body weight and sex ratio differences between both dietary groups were assessed by means of the Student's *t*-test. Male and female body weight differences within each age, diet, and family (or grouped families) were determined by means of the Student's *t*-test. One-way ANOVA, followed by a Holm-Sidak *post-hoc* test, was conducted in order to assess significant differences in male-to-female sex steroids (11-KT and E_2_) concentration between families of the same diet and age. Sex steroid differences between diets within each family were assessed by means of a Student's *t*-test. For evidencing gradation in sex steroids and body weight between diets, families, and males/females of each family, a principal component analysis (PCA) was performed using EZinfo version 3.0 (Umetrics, Umea, Sweden). Differences in E_2_/11-KT quotient between families and diets were assessed by means of a one-way ANOVA, followed by a Holm-Sidak *post-hoc* test.

## Results

### Growth and sex ratio progression

**Figure 1:**
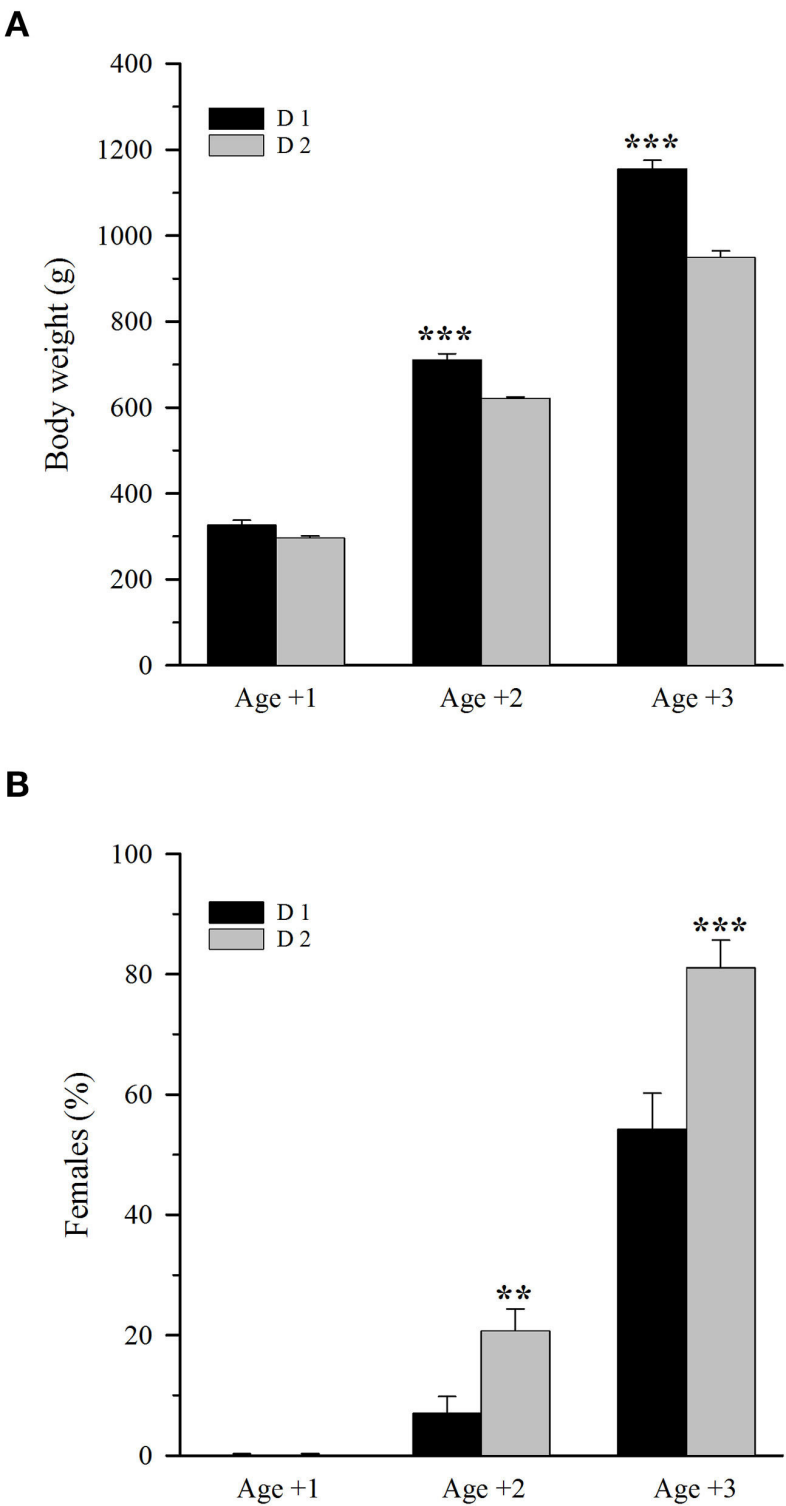
**(A)** Body weight and **(B)** female percentage in 1-, 2-, and 3-year-old gilthead sea bream fed an FM/FO diet (D1) or a plant-based diet (D2). Values are the mean ± SEM of three tanks per diet (*n* = 261 (D1) −271 (D2) fish/diet at Age +1, 202 (D1) −207 (D2) fish/diet at Age +2, 138 (D1) −146 (D2) fish/diet at Age +3). Asterisks indicate significant differences between the experimental diets within each age (Student's *t*-test, **P < 0.01, ***P < 0.001).

Dietary treatment had a clear effect on fish size regardless of their genetic background. Thus, considering all fish families as a whole under each dietary treatment, fish fed D2 consistently showed a lower body weight than that fed D1. Differences in body weight were not statistically significant at Age +1 (Figure 1A). However, at Age +2 and Age +3, the body weight difference between both dietary groups was 12.5 and 18%, respectively, with the growth performance negatively affected (*P* < 0.001) by the plant-based diet. Regarding sex, all fish were males at Age +1 regardless of diet (Figure 1B). However, the plant-based diet largely enhanced the male-to-female sex reversal, resulting in a significantly (*P* < 0.01) higher female percentage at both Age +2 (20.9 vs. 7.2%) and Age +3 (81.2 vs. 54.4 %).

### Sexual dimorphism: Body weight and sex steroids

**Figure 2:**
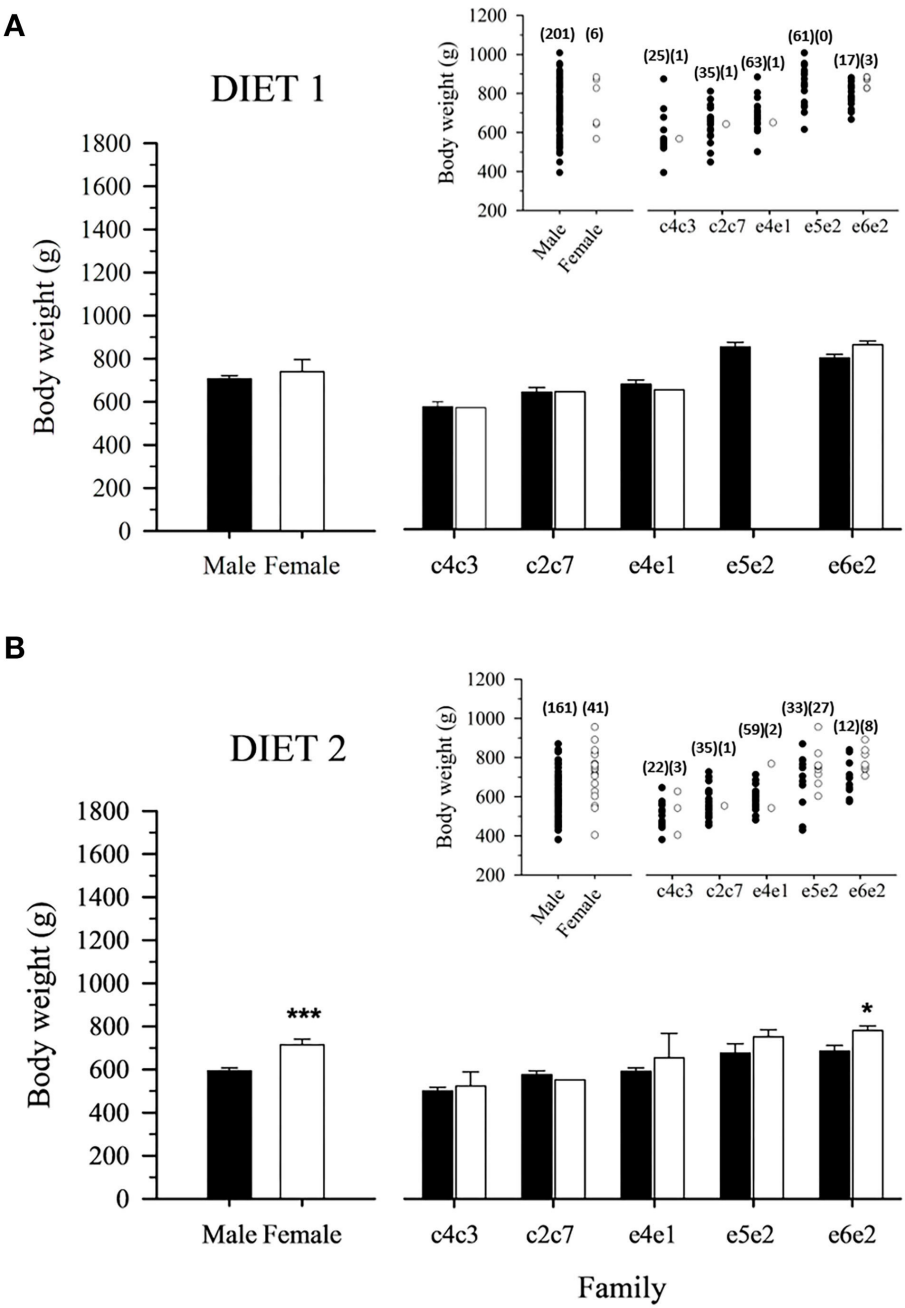
Mean male and female body weight of 2-year-old gilthead sea bream-fed control **(A)** or experimental diet **(B)** as a whole population and separated by family. Values are the mean ± SEM of three tanks per diet (*n* = 207 fish for diet 1, 202 for diet 2). Asterisks indicate significant differences (Student's *t*-test, *P* < 0.05) between males and females body weight. Inserts indicate individual body weight in each population. The number of males and females for the populations and families is indicated in parenthesis.

Data on body weight at Age +2 showed no significant differences between males and females fed D1, neither when considering all fish families as a whole nor analyzing each family separately (Figure 2A). However, in fish fed D2, a significant body weight sexual dimorphism toward larger females was evidenced comparing male and female populations (Figure 2B). It must be noted that this feature was significant only for the fast-growing family e6e2 (Figure 2B).

**Figure 3:**
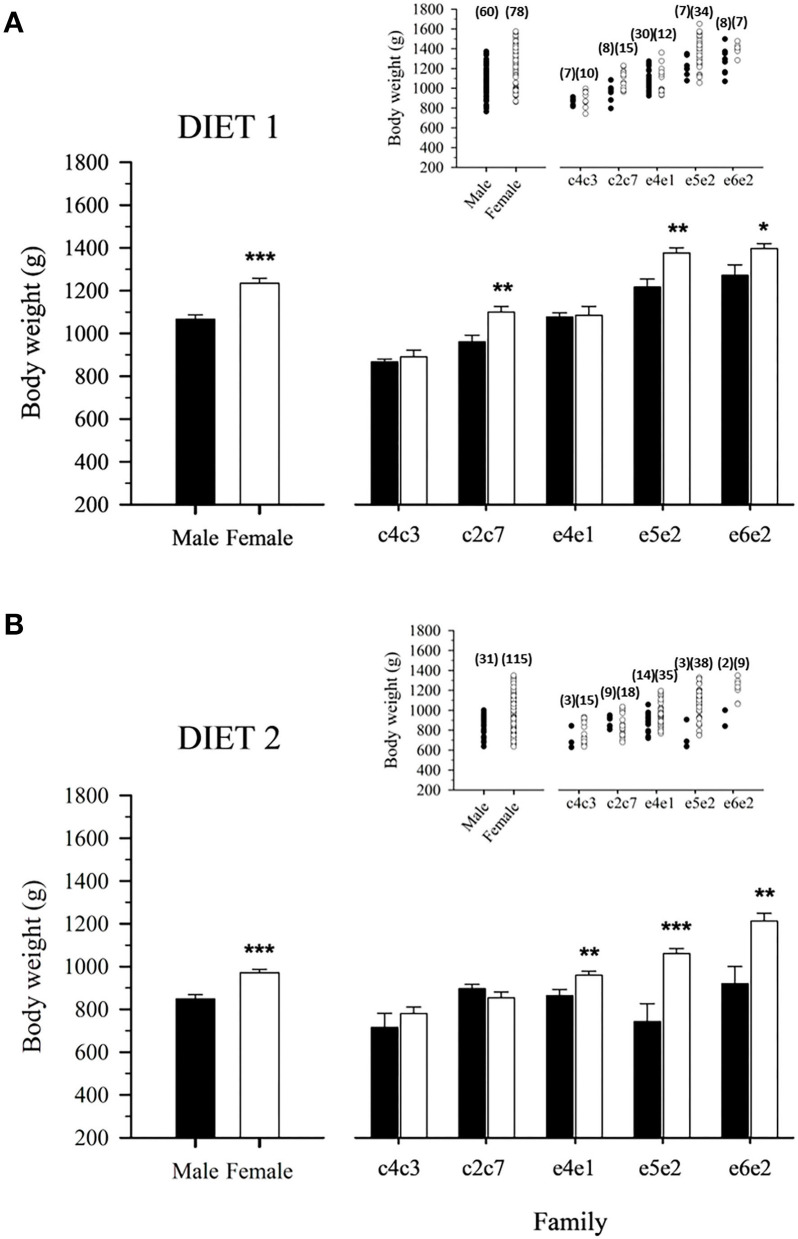
Mean male and female body weight of 3-year-old gilthead sea bream-fed control **(A)** or experimental diet **(B)** as a whole population and separated by family. Values are the mean ± SEM of three tanks per diet (*n* = 138 fish for diet 1, 146 for diet 2). Asterisks indicate significant differences (Student's *t*-test, *P < 0.05, **P < 0.01, ***P < 0.001) between males and females body weight. Inserts indicate individual body weight in each population. The number of males and females for the populations and families is indicated in parenthesis.

At Age +3, gilthead sea bream females had approximately 15% more body weight than their male counterparts regardless of diet (Figure 3). In fish fed D1, this clear sexual dimorphism was mostly observed in families c2c7, e5e2, and e6e2 (Figure 3A), whereas for fish fed D2, it was supported by a significantly higher body weight of females of families e4e1, e5e2, and e6e2 (Figure 3B).

**Figure 4:**
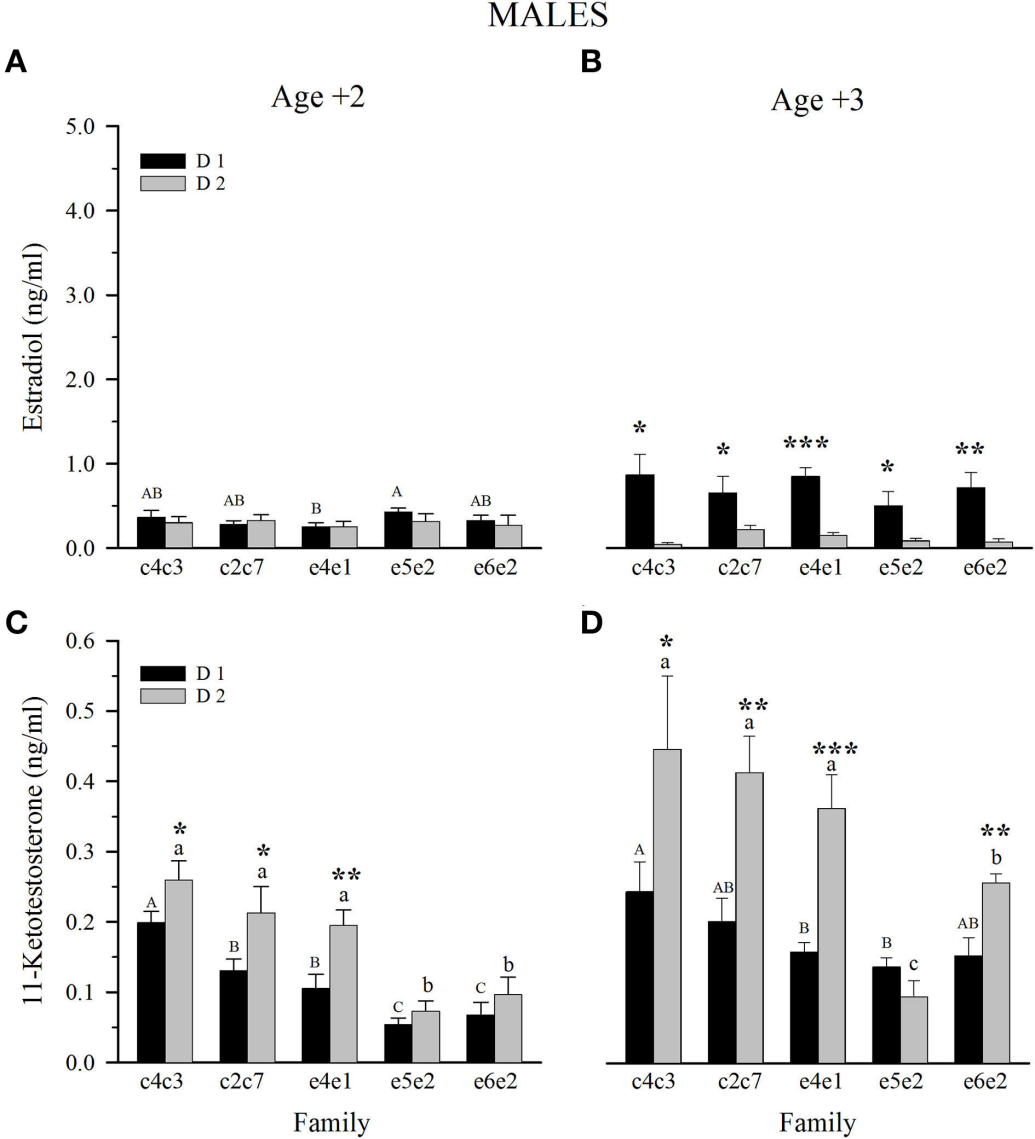
Plasma levels at spawning season of estradiol **(A, C)** and 11-ketotestosterone **(B, D)** in 2 and 3-year-old males fed control (D1) or experimental diet (D2). Values are the mean ± SEM. Different capital letters indicate significant differences between families fed D1. Different lowercase letters indicate significant differences between families fed D2. Asterisks indicate significant differences between dietary groups within each family (Student's *t*-test, *P < 0.05, **P < 0.01, ***P < 0.001).

E_2_ plasma levels in Age +2 males were quite similar, with the only significant difference between families e5e2 and e4e1 when fed the D1 diet (Figure 4A). For Age +2 and Age +3, plasma levels of E_2_ increased around 130% in males fed D1, whilst those of males fed D2 decreased to around 50% (Figure 4B). Age +3 male fish fed D1 showed higher E2 plasma levels than fish fed D2, with no differences among families for the same dietary group. Male plasma levels of 11-KT at Age +2 showed a gradually decreasing trend from slow- to fast-growing families, with e5e2 and e6e2 families significantly different from the slow and intermediate families within both diets (Figure 4C). Comparison between diets showed that fish fed D2 had significantly higher levels of 11-KT in the case of slow- and intermediate-growing families, and the same trend, although non-significant, was maintained in fast-growing families (Figure 4C). Male 11-KT levels generally increased from Age +2 to Age +3, keeping the gradual decrease of 11-KT from slow- to fast-growing families (Figure 4D). Four of five families of Age +3 males displayed higher 11-KT levels when fed D2.

**Figure 5:**
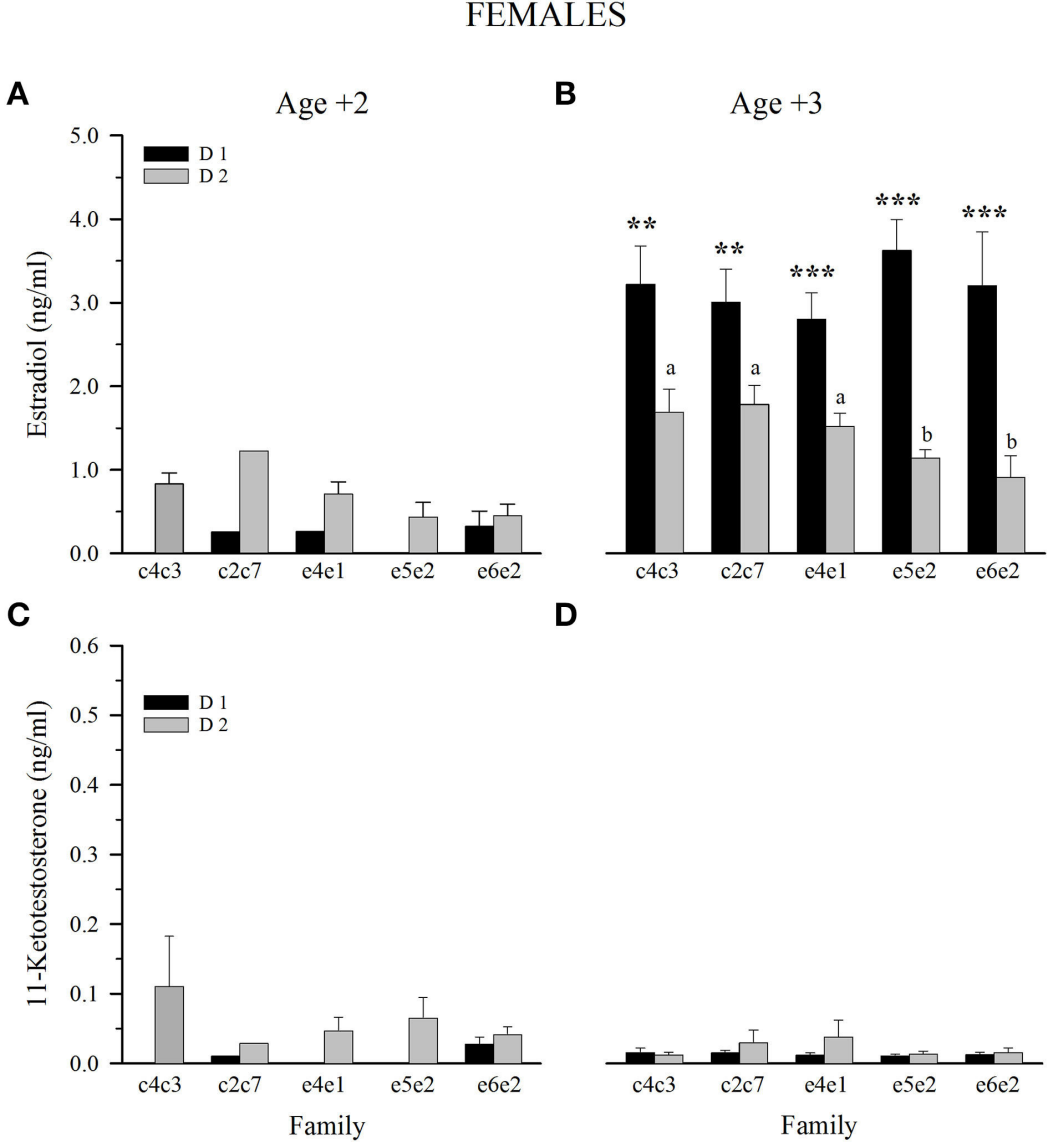
Plasma levels at spawning season of estradiol **(A, C)** and 11-ketotestosterone **(B, D)** in 2 and 3-year-old females fed control (D1) or experimental diet (D2). Values are the mean ± SEM. Different letters indicate significant differences between families fed D2. Asterisks indicate significant differences between dietary groups within each family (Student's *t*-test, **P < 0.01, ***P < 0.001).

Results of female sex steroids at Age +2 were not conclusive due to the low number of gilthead sea bream that underwent sex change from male to female (Figures 5A, C). Nonetheless, at Age +3, female E_2_ plasma levels showed a clear diet effect, with fish fed D1 having significantly higher levels within all families (Figure 5B). A genetic effect was reduced to fish fed D2, with higher circulating levels of E_2_ in slow-growing fish than in fast- and intermediate-growing families (Figure 5B). 11-KT plasma levels of all 3-year-old female families were below 0.05 ng/ml, and no differences were observed between families and dietary groups (Figure 5D).

### Sex steroids ratio

**Figure 6:**
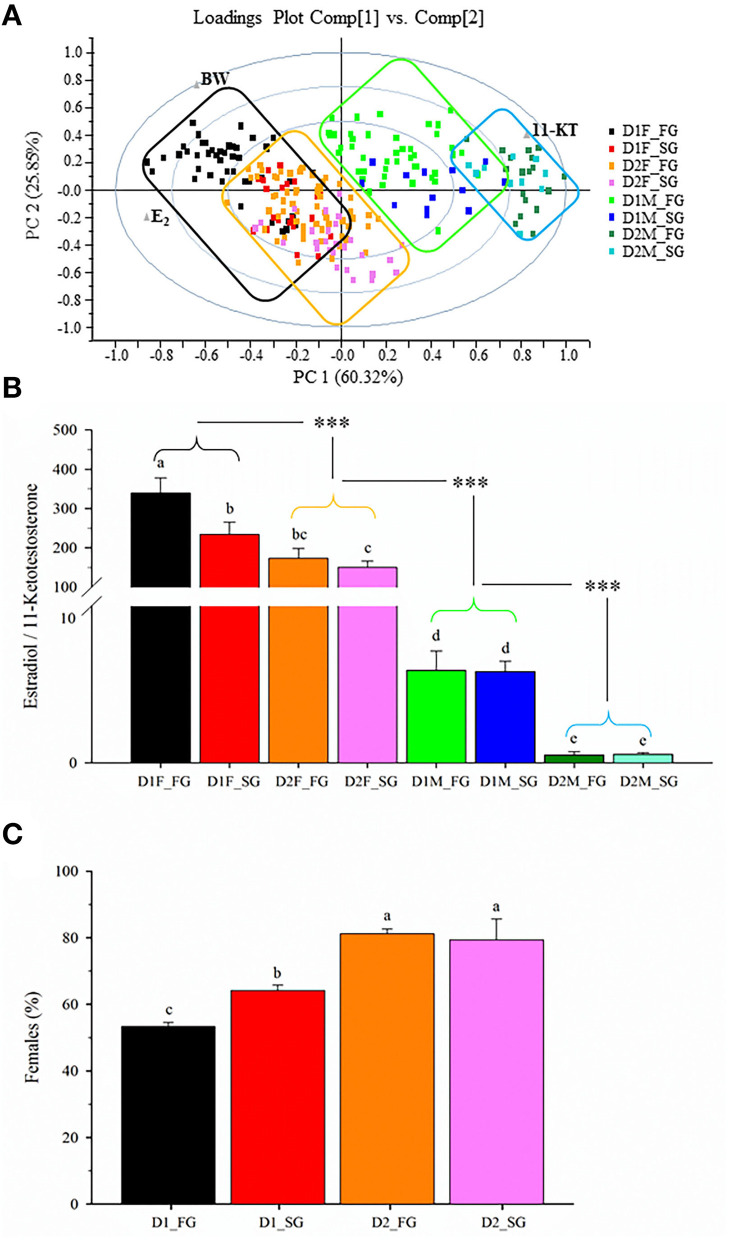
**(A)** Principal component analysis of body weight and plasma sex steroids levels during the second spawning period (3-year-old fish). **(B)** Sex steroids quotient of females (F) and males (M) of each dietary group (D1–D2), and fast (FG) or slow (SG) growth family groups. Values are the mean ± SEM of the E_2_/11-KT quotient. Different letters indicate significant differences between each group. Asterisks indicate significant differences between males and female groups with different dietary regimes (Holm-Sidak *post-hoc* test, ***P < 0.001). **(C)** Female percentage of fast and slow growth family groups.

The PCA of plasma sex steroid levels at Age +3 showed that >86% of the total variance was explained by the two first components (Figure 6A). Each fish was categorized according to its diet, sex, and family group. For better representation, e_x_e_y_ families were joined as a unique fast-growth family group, while c_x_c_y_ families were joined as a unique slow-growth family group. Movement along the X-axis (60.32% of total variance) accounted for plasma sex steroid levels, with the highest values of E_2_ on the left and the maximum values of 11-KT on the right. This sex steroid distribution clearly discriminated females (black and orange boxes) on the left and males (green and blue boxes) on the right. The Y-axis (25.85% of total variance) accounted for body weight changes, separating the fast-growth families at the top from the slow-growth families at the bottom. In other words, the resulting plasma E_2_/11-KT ratio was affected by both diet and genetics, increasing this hormonal quotient with the improvement of both the nutritional and genetic background (Figure 6B). In males, a genetic effect was not seen, but the diet effect persisted with a decreased E_2_/11-KT ratio in fish fed the plant-based diet. This occurred in parallel with a genetically regulated male-to-female sex reversal, displaying fast-growing families fed D1 a significantly lower percentage of phenotypic females (54%) than slow-growing families (65%) fed the same diet (Figure 6C). This percentage of phenotyped females reached a plateau (79–81%) in fish fed D2 regardless of their genetic background, which is indicative of a genetic and nutrition interaction according to which the plasma E_2_/11-KT ratio becomes more fine-regulated in females than in males, and in fish fed D1 diet rather than in fish fed D2 diet. Moreover, it is noteworthy that the highest plasma E_2_/11-KT ratio (powerful sex female steroid profile) was concurrent with a lower abundance of functional/powerful females when fish from fast- (e_x_e_y_) or slow-growth (c_x_c_y_) fish families were grouped and analyzed together as two different experimental groups in our common garden rearing system.

## Discussion

The present study underlines the effect of different nutrition and genetic backgrounds in the plasma sex steroids profile and male-to-female sex reversal in gilthead sea bream with differences in heritable growth and a sexual growth dimorphism, which was exacerbated in fast-growing fish by feeding a plant-based diet. Indeed, when fish attained 2 years of age, a sexual growth dimorphism was only observed in fish fed D2. However, body weight differences intensified as fish grew, and were equally significant and visible with both diets, especially in fast-growing families. Besides, previous studies have highlighted that families with a fast growth phenotype within the PROGENSA^®^ selection program displayed a plastic gut microbiota to cope better with changes in diet composition, also contributing to a better disease progression of parasitic enteritis in fish challenged with the myxozoa *Enteromyxum leei* (5, 43). Available studies also evidenced that such selective breeding for growth and skeletal deformities has an impact on humoral immune markers (48), carcass and morphometric traits (49, 50), energy partitioning between growth and swimming activity (44), and even more in reproductive success as recently evidenced in fish with a normal phenotype but with a genetic background of skeletal deformities (51). It appears, thereby, that selection for growth and deformity traits co-selects in the PROGENSA^®^ selection program for a number of relevant traits, including the sex ratio, which is becoming nutritionally and genetically regulated through the life cycle. The ultimate physiological mechanism remains elusive, but this study aimed to provide new insights into the sex ratio in a protandrous fish for its use as an operational welfare scoring system in a challenging environment.

Sex reversal in fish is defined as a mismatch between the phenotypic and the genetic sex (52). Thus, in gilthead sea bream, in particular, the general thinking is that individuals of this fish species act as functional males by the end of the first-second year of life, and sex reversal generally takes place 1 year after the first male sexual maturation (53). The percentage of male-to-female sex reversal can vary from 15 to 80% during the second year of life (53), but the possibility of a later sex reversal was reported by Brusléa-Sicard and Fourcault (54) and further corroborated by Chaoui et al. (55). Herein, the male-to-female sex reversal was accomplished by a relatively high percentage of individuals (50–85%) regardless of diet, which confirms the notion that a sex change in gilthead sea bream is cued by social and environmental factors when a critical age or size is attained (30, 56, 57). Indeed, the removal of functional females from the population drives the feminization of the remaining males (53, 58), probably *via* the production and release of specific pheromones that can activate or block some sex-specific networks. The aquatic environment may also contain a wide range of endocrine-disrupting chemicals that reduce reproductive performance and can even inhibit the sex reversal in gilthead sea bream (31, 59, 60). However, xenobiotic-induced sex reversal did not appear to be our case, because fish grew from early life stages in an eco-friendly environment where a wide-screening of undesirable compounds in fish edible matter revealed bio-contaminant loads to be much lower than the maximum established residue level (61).

The net balance between gonadal estrogen and androgen production directs sexual differentiation and gonadal development in fish (62). Indeed, E_2_ and 11-KT are typically considered the predominant steroids in the regulation of sex change in most fish species (63). Thus, we found herein that both E_2_ and 11-KT increased over time, with males and females displaying the highest plasma levels of 11-KT or E_2_ at Age +3, respectively. A nutritionally mediated effect was also reported, although it is difficult to deconvolute the extent to which this observation is due to a specific nutrient or to a different loading of plant phytoestrogens with both estrogenic and antiestrogenic effects on vertebrates (64). In tilapia farming, in particular, herbal extracts could be used as safe alternative agents to control precocious tilapia maturity and prolific breeding in production (65). In the present study, the inclusion level of soy protein concentrate, a rich source of phytoestrogens, varied between 16% in D1 and 25% in D2, although it is within the tolerance range for gilthead sea bream (65). In any case, plasma levels of fish fed D2 were lower for E_2_ and higher for 11-KT, displaying these fish a masculinized sex steroid profile that would promote the male-to-female sex reversal in the absence of high powerful functional females that ensure reproduction success, as stated before by Simó-Mirabet et al. (41). Masculinization of gonochoristic fish populations also occurs as a result of elevated temperatures and other environmental stressors. This would be mediated, at least in part, by the increase of circulating cortisol, which is now recognized as a universal mediator of sex reversal in fish due to its implication in delaying ovarian meiosis and increasing 11-KT (52). For instance, in the protogynous three-spot wrasse, cortisol treatment had a masculinizing effect (66). This feature was also reported by us in the protandrous gilthead sea bream-fed plant-based diets, regardless of the well-known hypocholesterolemic effect of plant ingredients in most farmed fish (67). In fact, since cholesterol is the precursor of cortisol, its reduced dietary supply or intestinal absorption could initially lead to a female-biased sex ratio. Nonetheless, there are more factors at play in this process, and Nile tilapia fry fed with saponin-supplemented diets (hypocholesterolemic diets) displayed a significant male-biased population (68). In other words, the sex ratio can be influenced by a number of nutritional factors, including changes in the dietary fatty acid composition as a result of a high replacement of marine feedstuffs by vegetable oils (69). However, all this is the result of a complex trade-off, which is also indicative of the amazing diversity and evolution of sex determination in vertebrates, and fish in particular (70). Similarly, global warming due to climate change would affect the offspring quality of a wide range of animals (71–73). This is especially important for aquatic ectotherms, where temperature values above the optimal for each species and fish strain can shift the sex ratio toward either the male or female phenotype in gonochoristic fish (74–76) and perhaps hermaphroditic fish.

Moreover, in the present study, discriminant analysis of sex steroids and body weight displayed a clear separation of phenotypic males and females, with also a differentiation of slow- and fast-growth families. Thus, families of fast heritable growth displayed a more mature/advanced sex steroid phenotype, resulting in a higher E_2_/11-KT ratio (feminization phenotype) that would trigger the inhibition of male-to-female sex reversal of the remaining males. This hormonal quotient, rather than the circulating amount of a given sex steroid, would determine what sex-specific network is activated or suppressed, leading or not to the sex reversal in the individual. Indeed, the rise of the E_2_/11-KT ratio was associated with a female bias in painted turtles (77), and we found herein that this plasma sex steroid ratio was progressively increased from males to females with both the nutritional and genetic improvement. However, the increased plasma E_2_/11-KT ratio in female fish fed D1 was negatively associated with the rate of male-to-female sex reversal in genetically improved fish, whereas this genetically mediated response was mostly masked in fish fed D2. This finding highlighted a nutrition and genetic interaction on the progression of sexual maturation, as reported for gut microbiota composition and function. Indeed, fast-growing fish families in the PROGENSA^®^ program are more resilient to changes in gut microbiota composition, but at the same time, metatranscriptomic analyses confirm and extend the notion that the core microbiota of genetically improved fish is able to modulate their metabolic activity to cope better with changes in diet composition (5, 43). Otherwise, there is no evidence in gilthead sea bream that the modulating effects of gut microbiota by feed additives are a specific feature of each additive and genetic background (unpublished results).

As indicated earlier, reproductive physiology and spawning are sensitive processes to changes in environmental conditions and physiological stress, and how and to what extent external and internal factors have an impact on broodstock welfare is of relevance to assure reproduction success, but also the offspring plasticity and quality (78, 79). Concretely, in gilthead sea bream, several attempts at nutritional programming are made through changes in the parental nutrition (80–83). Such an approach is not unique to fish, as parental nutrition and hormonal status of humans and terrestrial animals directly impact all stages of gamete maturation, fetal development, and long-term offspring health (84, 85). Therefore, one of the challenges of modern aquaculture is to assure the welfare of breeders, which could also inform the welfare condition from a retrospective point of view. Certainly, we can conclude that fast-growing fish families fed a control diet became powerful females, whilst fish of slow-growth families and/or fish fed plant-based diets experienced a pseudo-feminization effect (i.e., fish with a weaker female signal, which enhances the ratio of sex reversal). Therefore, it appears that the progression of sex reversal is directly regulated by both nutritional and genetic background among many other environmental and social factors. Thus, the study of sex reversal as a biological endpoint is becoming a reliable tool of relevance for the animal welfare certification of a highly cultured protandrous fish such as gilthead sea bream. Several items support this assumption. First, fish growing with plant-based diets from early life stages shared an enhanced onset of puberty and sex reversal in concurrence with some growth impairment of sexually mature fish. Second, genetically improved fish for growth are more resilient to the progression of male-to-female sex reversal with the use of alternative fish feed formulations, but further research should be directed toward the effect of specific nutrients on reproductive performance and maturation as a means to enhance the offspring quality. Finally, a decreased plasma E_2_/11-KT ratio is becoming indicative of a negative welfare status in the long term, supporting this finding the use of such reproductive tract scoring systems for leading a protandrous farmed fish toward their optimum welfare condition.

## Conclusion

Results of this long-term dietary and genetics trial disclose that sex steroids profile and male-to-female sex reversal are nutritionally and genetically regulated in the protandrous gilthead sea bream. Moreover, the sex ratio is proposed as a reliable welfare indicator alerting of disturbances in reproductive performance, and perhaps overall growth and offspring quality. Such a scoring system is becoming, thereby, an exploitable finding for the certification of animal welfare in a given gilthead sea bream production system.

## Data availability statement

The raw data supporting the conclusions of this article will be made available by the authors, without undue reservation.

## Ethics statement

The animal study was reviewed and approved by Ethics and Animal Welfare Committee of the Institute of Aquaculture Torre de la Sal and CSIC Ethics Comittee.

## Author contributions

JP-S: conceptualization. PH, AF, JC-G, and JP-S: formal analysis. AF, JC-G, and JP-S: funding acquisition. PH, AF, and JP-S: methodology. AF, JA, and JP-S: resources. PH, JC-G, and JP-S: draft writing. PH, AF, JC-G, JA, and JP-S: draft review and edition. All authors contributed to the article and approved the submitted version.
