# Supplementary material for: Use of male-to-female sex reversal as a welfare scoring system in the protandrous farmed gilthead sea bream (Sparus aurata)
Source: Front Vet Sci. 2023 Jan 9;9:1083255. doi: 10.3389/fvets.2022.1083255 (PMC9868933; doi:10.3389/fvets.2022.1083255)
Supplement: Supplementary file 1 [file Table_1.DOCX]

**Supplementary Table 1.** Ingredients and chemical composition of experimental diets.

| Ingredient (%) | D1 |  | D2 |
| --- | --- | --- | --- |
|  |  |  |  |
| Fish meal | 23.0 |  | 3.0 |
| Fish hydrolysate (CPSP) | 2.0 |  | 2.0 |
| Soya protein | 16.7 |  | 25.6 |
| Rapeseed cake | 12.0 |  | 10.0 |
| Wheat gluten | 4.5 |  | 7.3 |
| Corn gluten | 16.5 |  | 25.5 |
| Wheat | 10.0 |  | 7.4 |
| Fish oil | 14.1 |  | 3.9 |
| Rapeseed oil | 0 |  | 9.0 |
| Mineral-vitamin mix^a^ | 1.26 |  | 6.3 |
|  |  |  |  |
| Proximate composition (%) |  |  |  |
| Moisture | 7.9 |  | 7.5 |
| Crude protein | 45.0 |  | 45.0 |
| Crude fat | 20.1 |  | 20.1 |
| Ash | 6.9 |  | 5.9 |
| NFE^b^ | 19.1 |  | 19.8 |
| ARA^c^ | 0.17 |  | 0.05 |
| EPA^d^ | 2.30 |  | 0.60 |
| DHA^e^ | 1.50 |  | 0.42 |
| EPA+DHA | 3.80 |  | 1.02 |
| Crude energy (MJ/kg) | 22.1 |  | 22.3 |

^a^ Contains vitamins, minerals, amino acids, cholesterol, lecithin and anti-oxidants.

^b^ Nitrogen free extract.

^c^ Arachidonic acid (20:4n-6).

^d^ Eicosapentaenoic acid (20:5n-3).

^e^ Docosahexaenoic acid (20:6n-3).
